# Mass separator for radioactive isotopes

**DOI:** 10.1007/s10967-017-5649-4

**Published:** 2018-01-05

**Authors:** Anthony D. Appelhans, John E. Olson, David A. Dahl, Michal B. Ward, Troy A. Robinson, James E. Delmore

**Affiliations:** Idaho National Laboratory, 2525 N. Fremont Avenue, 83415 Idaho Falls, ID USA

**Keywords:** Isotope separator, Radioactive xenon standards, Ion beam intensity

## Abstract

A new isotope separator has been designed, constructed, and put into routine operation for separation of ^133^Xe providing a major advancement and significant cost reduction in preparation of this radioactive isotope. The design features and advantages are discussed that expedite high purity separation of relatively small quantities of this isotope. These advantages could be easily used to expedite separation of other shorter-lived radioactive isotopes.

## Introduction

Idaho National Laboratory (INL) has long supported nonproliferation monitoring by supplying radioactive xenon standards to laboratories [1]. One aspect of this support is the production of radioactive ^133^Xe (5.423 day), which is free from all other detectable radioactive species. The most practical method for producing this isotope is by fission, a process that also produces the radioactive isotopes ^131m^Xe (11.9 day), ^133m^Xe (2.19 day), and ^135^Xe (9.1 h). ^135^Xe and ^133m^Xe have shorter half-lives and can be allowed to decay prior to use. In contrast, the longer half-life of ^131m^Xe allows the ratio of ^131m^Xe–^133^Xe to increase with time; hence, the requirement for separation.

Historically, INL produced this species by isotope separation of fresh fission product gases using an aging isotope separator originally designed for the separation of weighable quantities of multiple isotopes [2]. A much smaller isotope separator has been designed, constructed, and put into routine use that takes advantage of the fact that only small quantities of a single radioactive isotope are required. This approach allowed major simplifications while improving efficiency by several orders of magnitude to the point where medical quantities of this isotope are now used as the starting material. This instrument is now in routine production of this isotope.

An isotope separator designed for the production of multiple isotopes in weighable quantities has a number of requirements that include:

1. An ion source capable of producing milliampere ion beams.
2. An accelerating voltage sufficiently high (~ 40 kV) to reduce ion transit time, which in turn prevents excessive beam expansion from charge repulsion within the high intensity ion beam.
3. A magnet and flight tube sufficiently wide to accommodate multiple masses without wiping the sides of the flight tube.
4. A collector assembly capable of capturing all of the desired isotopes while minimizing self-sputtering from the collectors.

With much smaller quantities of a single isotope, these requirements are greatly simplified. The ion beam intensity is reduced by several orders of magnitude, thereby reducing charge-repulsion-driven beam expansion. Lower ion beam intensity also allows for a lower ion source voltage, which in turn reduces arcing. The flight tube and magnet requirements are reduced as well, allowing these components to be much smaller.

The collector can be simplified by shuttling unwanted isotopes into beam dumps, while the single isotope of interest can be dispersed across a wider area to reduce self-sputtering. This is significant since Xe is a gas that must be implanted into a foil. With the reduced ion energy of the smaller instrument, it is necessary to float the collector at a negative voltage, thereby enhancing the depth of implant that in turn ensures the gas will not be sputtered back out of the foil [3, 4]. Floating a single collector is much simpler than floating the ion source at a higher energy; however, a floating collector functions as a de-focusing element, and thus the unwanted isotopes are sent to beam dumps prior to the slit that admits the isotope of interest into the floating collector. These features were incorporated into the new instrument.

## Instrument design and operation

An older VG 54 isotope ratio mass spectrometer originally configured with a thermal ionization mass spectrometry (TIMS) ion source was used as the basis of the instrument. The frame and magnet were retained without modification. The ends of the flight tube were reconfigured to better accommodate the additional components. The entire electronic package, ion source, ion collector, and pumping systems were replaced. The TIMS ion source was replaced with an electron bombardment ion source. An electrostatic dispersion lens [5] was added between the magnet and the collector, which widened both the dispersion and beam width without sacrificing resolution, reducing requirements for miniature high-precision components in the collector. This dispersion lens also served to spread the impact of the beam over a wider area to reduce self-sputtering of previously implanted Xe.

The ion source was designed with cylindrically focusing ion optics, thus providing a significant increase in ion transmission [6]. The high efficiency of the ion source allows high sample utilization allowing use of much smaller quantities of medical radioxenon.

One design downside of the cylindrical focusing ion lens is a reduction in peak shape quality and resolution when paired with the planar optics of the VG 54. However, the VG 54 was designed with sufficient resolution for actinide analysis; hence, the resolution was still more than adequate at this lower mass to provide unit resolution for xenon.

**Fig. 1:**
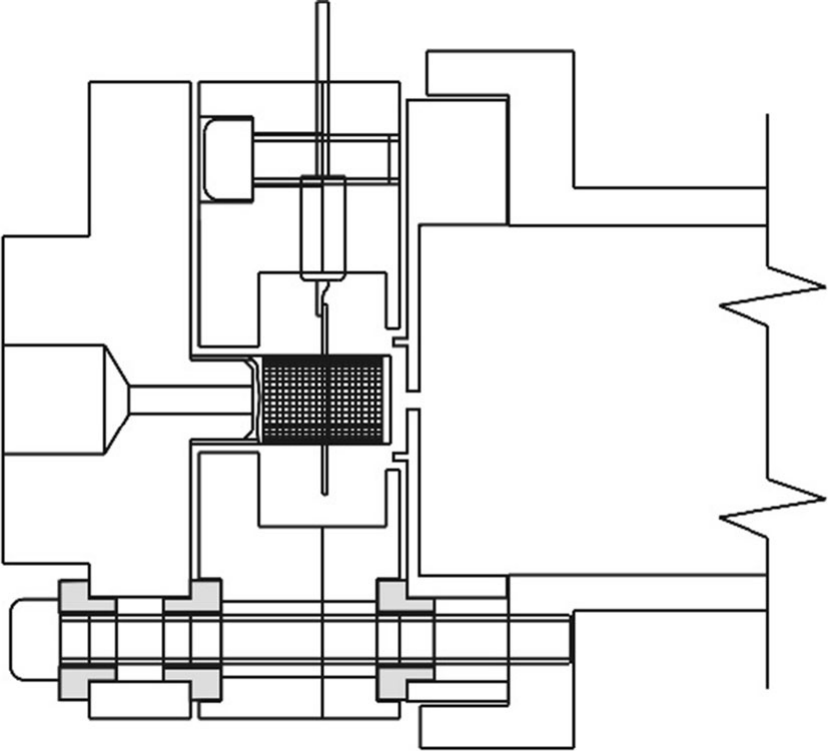
Drawing of the ion source and housing

Three identifiable regions are associated with the ion source: the gas handling system, the region with the actual ion source (see Fig. 1), and the ion drift region between the differential pumping aperture (that also serves as the focal point of the ion source) and the valve that allows isolation of the source from the magnet region.

The gas inlet housing has its own vacuum system consisting of a turbo pump, gauging, and a getter pump. The gas inlet system is connected to the ion source with a variable leak that can also function as a shut-off valve. This variable leak feeds a tube that passes the gas directly into the enclosed ion source, which in turn is inside the ion source housing so there is a higher pressure of Xe in the ion formation region.

The ion source and ion drift regions share a turbo pump and each has an isolation valve. This allows the region after the focusing aperture to continue being pumped while the ion source region is isolated from the turbo and only pumped through the focusing aperture. This configuration allows the focusing aperture to function as a differential pumping aperture, thereby reducing the rate of sample loss to the pumps.

Samples of medical ^133^Xe are received in glass ampoules with a rubber septum. This ampoule is placed in the gas inlet system with the septum opposite a needle. The vacuum system is then sealed and evacuated with the turbo pump. With the inlet still sealed from the source housing, a mechanism is activated to push the ampoule into the needle and release the Xe and carbon dioxide carrier gas into the sample inlet volume. A scan over both the CO–CO_2_ and Xe mass regions is conducted and a CO_2_/^129^Xe ratio calculated. Next, the getter pump is valved into the system that chemically reacts with the reactive gases to remove them from the system. A second scan over both the CO–CO_2_ and Xe mass regions is conducted and a new CO_2_/^129^Xe ratio is calculated. The use of the chemical getter typically results in a reduction of the CO_2_/^129^Xe ratio from ~ 10–15 down to < 0.1. When the appropriate vacuum is reached, the turbo pump is valved off. The getter pump removes the carbon dioxide and leaves relatively high purity sample of Xe. When the pressure stabilizes (typically ~ 1.0E − 05 Torr), the system is ready for separation of ^133^Xe.

### Performance

**Fig. 2:**
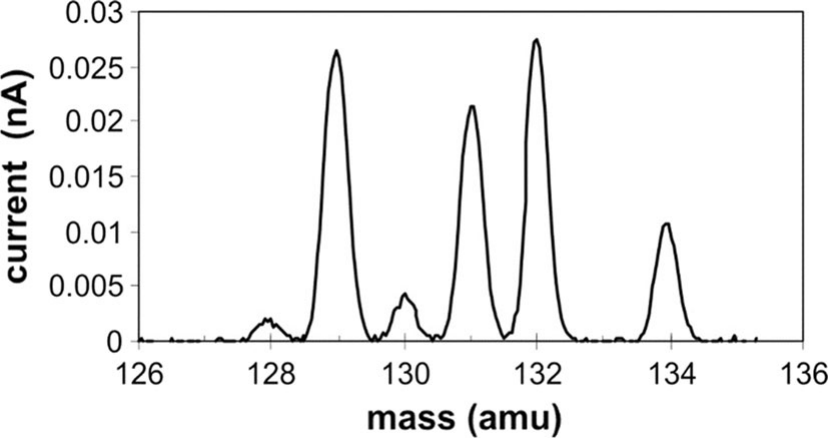
Spectrum of natural Xenon

Figure 2 is a scan of natural Xe collected on the Faraday cup adjacent to the implant collector. The peaks do not have flat tops because of a large cylindrical focusing aperture for high ion transmission and the combining of cylindrical and planar ion optics. This is not a problem since the purpose of the instrument is not to measure isotope ratios but to obtain a pure radioactive isotope in as high a yield as practical. Using the small (20 millicurie) medical source has greatly simplified the radiation control requirements.

In order to obtain an approximate collection efficiency, several samples were counted prior to separation and again after separation. Due to the fact that the starting sample was counted as a gas in a glass container and the separated sample was counted as being implanted in a metal foil, the geometries were not identical. However, the foil was shaped as closely as possible so as to minimize the geometry differences. These ratios averaged in the range of one part out of a thousand, or 0.10%.

Fission product Xe is obtained from a local secondary vendor who obtains the material from the Chalk River facility in Canada. Since the main application of radioxenon is for lung scans, efforts are made to obtain relatively large ratios of ^133^Xe/^131m^Xe in order to reduce longer-term exposure levels since ^131m^Xe has a much longer half-life than ^133^Xe. This is accomplished by extracting the gas within a relatively short time after irradiation. Since the 131 iodine precursor has an 8-day half-life while the 133 iodine precursor has a 20.8 h half-life, this is accomplished by rapid processing. Approximate ratios are in the range of 59, which indicates very rapid processing although this ratio decreases with time. ^131m^Xe is undetectable following isotope separation even after two months of decay time. Calculations using less than values for ^131m^Xe for an aged sample indicate that the final sample has a ratio of at least 29,000 ± 4000.

## Conclusion

The design and operability of a mass separator for collecting only one radioactive isotope at a time has been shown to be both economically and technically feasible. It was designed and constructed for a fraction of the cost of a large isotope separator that is capable of producing weighable quantities of multiple isotopes in a single run. The new instrument occupies a small corner of a lab while the original isotope separator occupied an entire room. Maintenance, operating, and radiation control expenses have been greatly reduced.
